# Infective Necrotizing Scleritis Post-percutaneous Sclerotherapy for Orbitofacial Venous Malformation

**DOI:** 10.7759/cureus.94726

**Published:** 2025-10-16

**Authors:** Zhen Ning Low, Nurul-Laila Salim, Rohanah Alias, Kavitha Saravanamuthu, Sharifah Intan Hosnaliza Syed Osman

**Affiliations:** 1 Ophthalmology, Kuala Lumpur Hospital, Kuala Lumpur, MYS

**Keywords:** complication of treatment, necrotizing scleritis, orbitofacial venous malformation, percutaneous sclerotherapy, sodium tetradecyl sulfate

## Abstract

Orbitofacial venous malformations (VMs) remain a challenge for the treating physician and often require a multidisciplinary treatment approach. Percutaneous sclerotherapy has been widely used as an alternative to surgical excision due to its favorable effectiveness and safety profile. We report a case of a patient with a known history of orbitofacial VM, previously treated with eight sessions of percutaneous sclerotherapy, who developed a rare complication following a ninth session involving sodium tetradecyl sulfate injection.

## Introduction

Orbitofacial venous malformations (VMs) are low-flow, congenital vascular anomalies within the orbit that can lead to symptoms such as proptosis, diplopia, or visual impairment. The management of orbitofacial VMs remains challenging due to their deep or intraconal location, infiltrative nature, and tendency to bleed during surgical intervention. Orbitofacial VMs are typically managed in a multidisciplinary setting involving both interventional radiologists and oculoplastic surgeons. Treatment modalities include conservative observation, percutaneous sclerotherapy, embolization, laser therapy, surgical intervention, and radiotherapy. Among these, image-guided percutaneous sclerotherapy has become a prominent first-line treatment, utilizing agents such as sodium tetradecyl sulfate (STS), absolute ethanol, or bleomycin. Among these treatment modalities, image-guided percutaneous sclerotherapy has gained increasing popularity due to its excellent efficacy and favourable safety profile, with reported success rates of 75-90% for achieving good-to-excellent patient outcomes [1]. This report details a rare but serious complication of infective necrotizing scleritis following percutaneous sclerotherapy. The rationale for this report is to highlight this severe ophthalmologic complication to increase clinical awareness. It aims to elucidate the critical importance of early detection, discuss potential pathophysiological mechanisms, and underscore the necessity for immediate and aggressive management to prevent permanent vision loss.

## Case presentation

A 38-year-old woman who was a known case of orbitofacial VM had undergone eight sessions of percutaneous sclerotherapy for the past 15 years. She had defaulted on her follow-up since the last session of percutaneous sclerotherapy in 2020 due to the COVID-19 pandemic. She presented to the emergency clinic with worsening proptosis and reduced vision of the right eye (RE) for one week. She did not report any eye pain, bleeding, double vision, or any worsening ptosis. She denied any history of ocular trauma.

**Figure 1:**
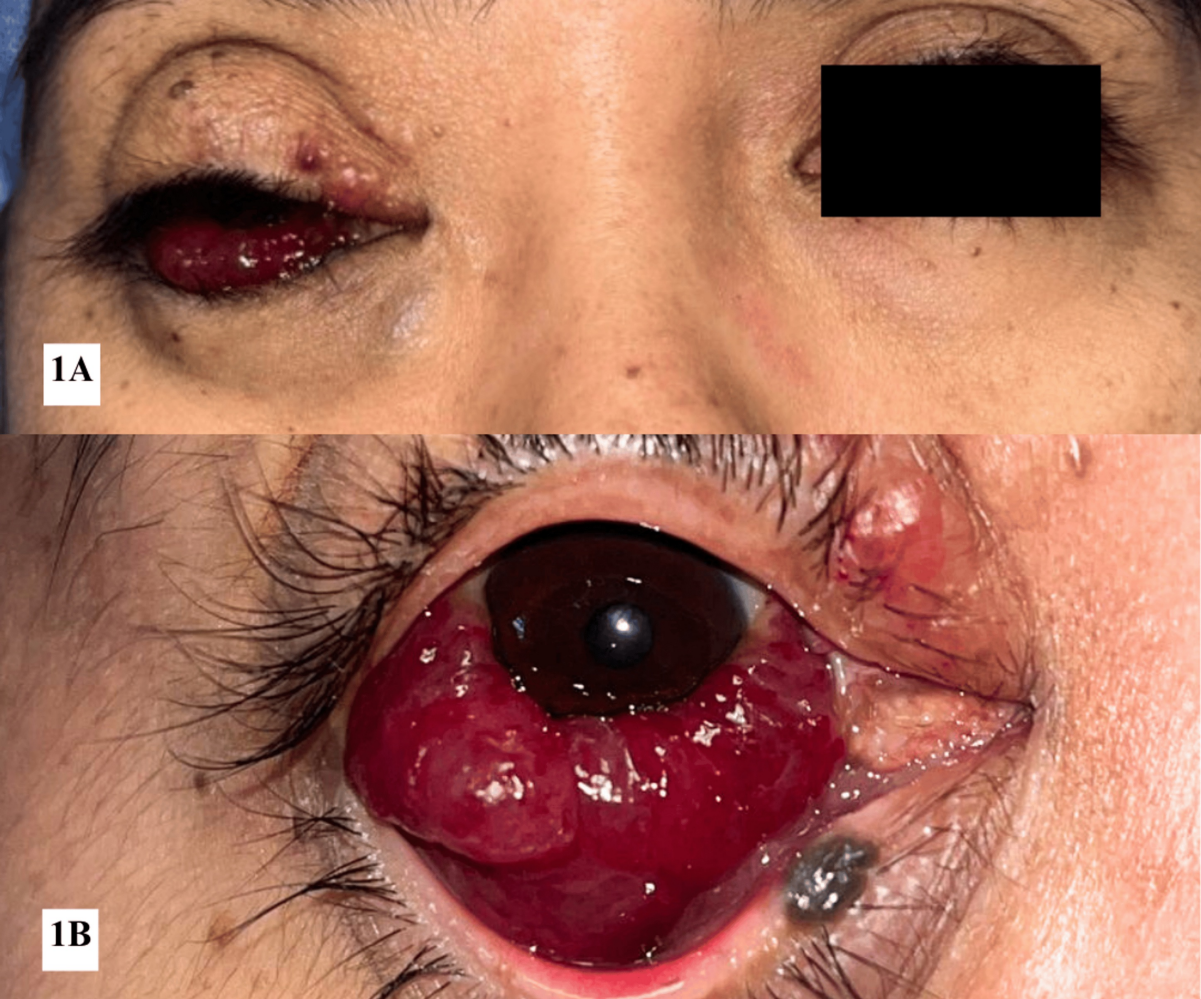
Right orbitofacial venous malformation, involving the right orbit, peri-orbital area, and cheek (1A) Swollen right upper lid covering the visual axis and ocular dystopia. (1B) RE extensive inferior conjunctival chemosis and hyperaemia with hemorrhage.

Examination revealed RE vision of 6/60 (improving to 6/24 with pinhole), swollen upper lid with mechanical ptosis covering the visual axis, ocular dystopia, and limited extraocular movement in all gazes (Figure 1A). There was no relative afferent pupillary defect. Hertel exophthalmometry demonstrated a 2 mm relative proptosis of the RE, which measured 15 mm compared to 13 mm in the left eye (LE). There was extensive reddish soft to firm non-tender non-compressible inferior conjunctival swelling (Figure 1B). No thrill or pulsation was noted, the size of the lesion did not increase with Valsalva manoeuvre, and no lymphadenopathy was present. Intraocular pressure was within normal limits. Funduscopy showed a healthy optic disc (cup-to-disc ratio 0.3) with a flat retina. Examination of the LE was unremarkable, with 6/9 uncorrected vision.

**Figure 2:**
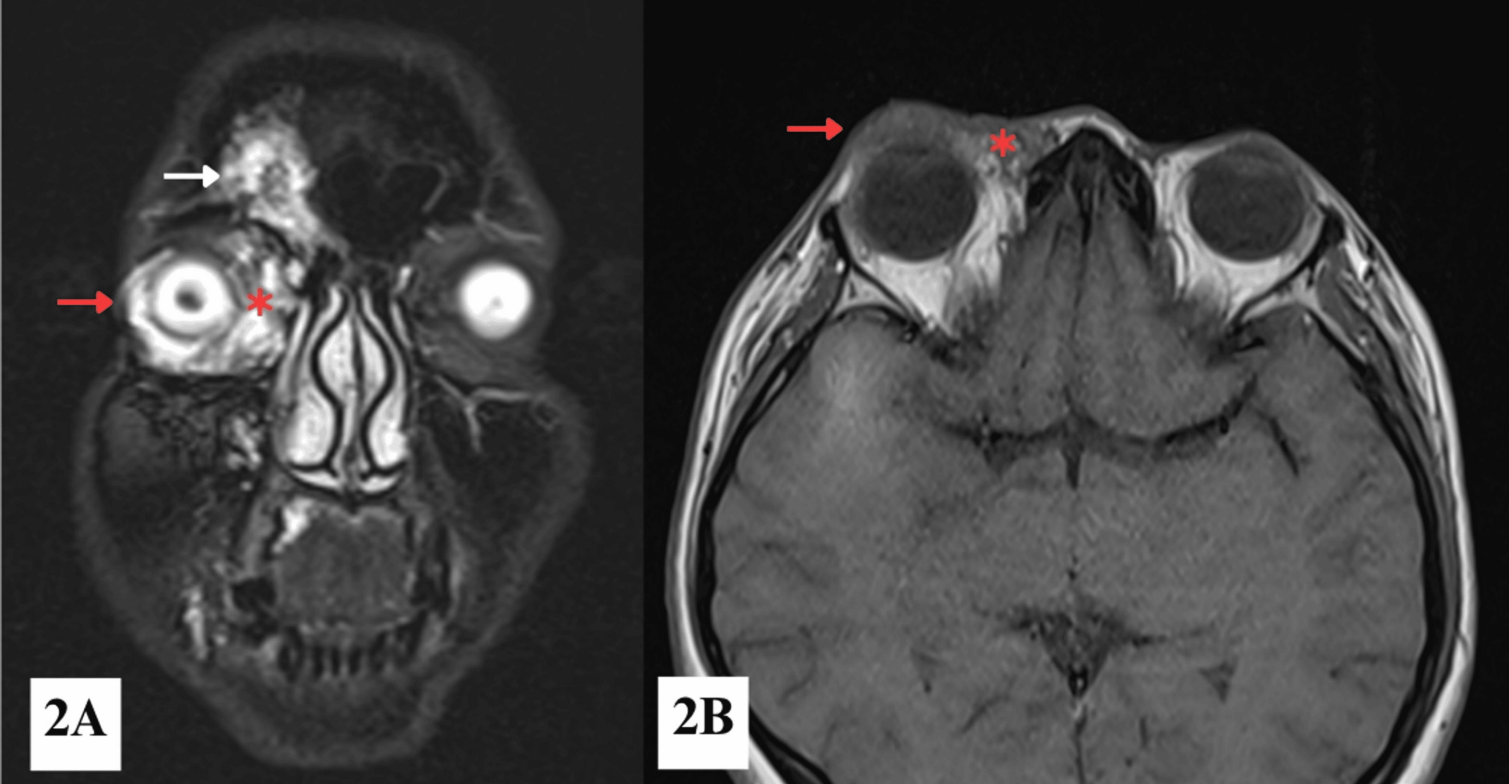
MRI showing (2A) a STIR coronal section of the face and (2B) a T1-weighted axial section of the orbit Multiple multilobulated lesions are seen involving the right frontal skull bone (white arrow), extending into the periorbital region and eyelid (red arrow), as well as the medial canthal area and the medial aspect of the right extraconal fat space (red asterisk).

Orbital and brain magnetic resonance imaging (MRI) revealed multiple large, irregular, multilobulated lesions over the right frontal skull, extending into the right frontal sinus, periorbital region, with involvement of the right lower eyelid, medial canthus, medial extraconal fat space, and orbital floor (Figure 2A). The largest lesion measured 1.4 x 3.4 x 2.3 cm (anteroposterior x transverse x craniocaudal) centered at the right lower eyelid. There was no intraconal extension. There was mild proptosis of the RE. Similar lesions were also noted at the frontal process of the right maxilla, extending to the hard palate, right maxillary tuberosity, alveolar process of the right maxilla, and right buccal space (Figure 2B). Time-of-flight (TOF) magnetic resonance angiography (MRA) showed slow-flow vascular malformations, likely venous in nature, with no evidence of arteriovenous malformation. Based on clinical and imaging findings, a diagnosis of right orbitofacial VM was confirmed. The patient was started on topical lubricants for symptomatic relief while awaiting image-guided percutaneous sclerotherapy.

Procedure for percutaneous sclerotherapy

**Figure 3:**
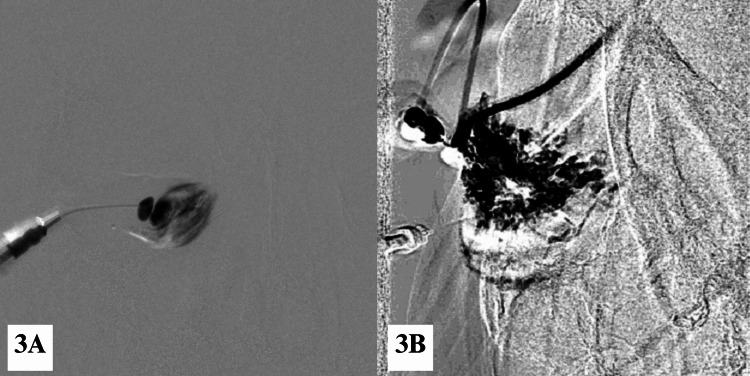
(3A) Early and (3B) late phases of percutaneous sclerotherapy for the right orbitofacial venous malformation by using a digital subtraction angiography technique

Percutaneous sclerotherapy was conducted under general anaesthesia with strict aseptic technique in an interventional radiology suite equipped with a biplane digital subtraction angiography (DSA) system. The procedure was done by an interventional radiologist with the assistance of an oculoplastic surgeon. Under real-time guidance with a high-resolution linear ultrasound probe (5-13 MHz), a 24-gauge branula was introduced percutaneously via the right inferior conjunctiva to access the venous malformation. Following successful cannulation, an attempt was made to aspirate the cyst contents. Subsequently, a sclerosant mixture comprising 3 mL of STS and Lipiodol in a 2:1 ratio was injected in small aliquots through the same puncture site (Figure 3A). The sclerosant was allowed to dwell within the lesion (Figure 3B). No extravasation was noted during the procedure. Haemostasis was achieved with manual compression, and no orbital bandaging was applied. The patient was returned to the inpatient ward for close monitoring. On post-sclerotherapy day one, the RE inferior conjunctiva appeared chemosed and less vascularised. Vision and extraocular movement remained unchanged. The patient was discharged home.

**Figure 4:**
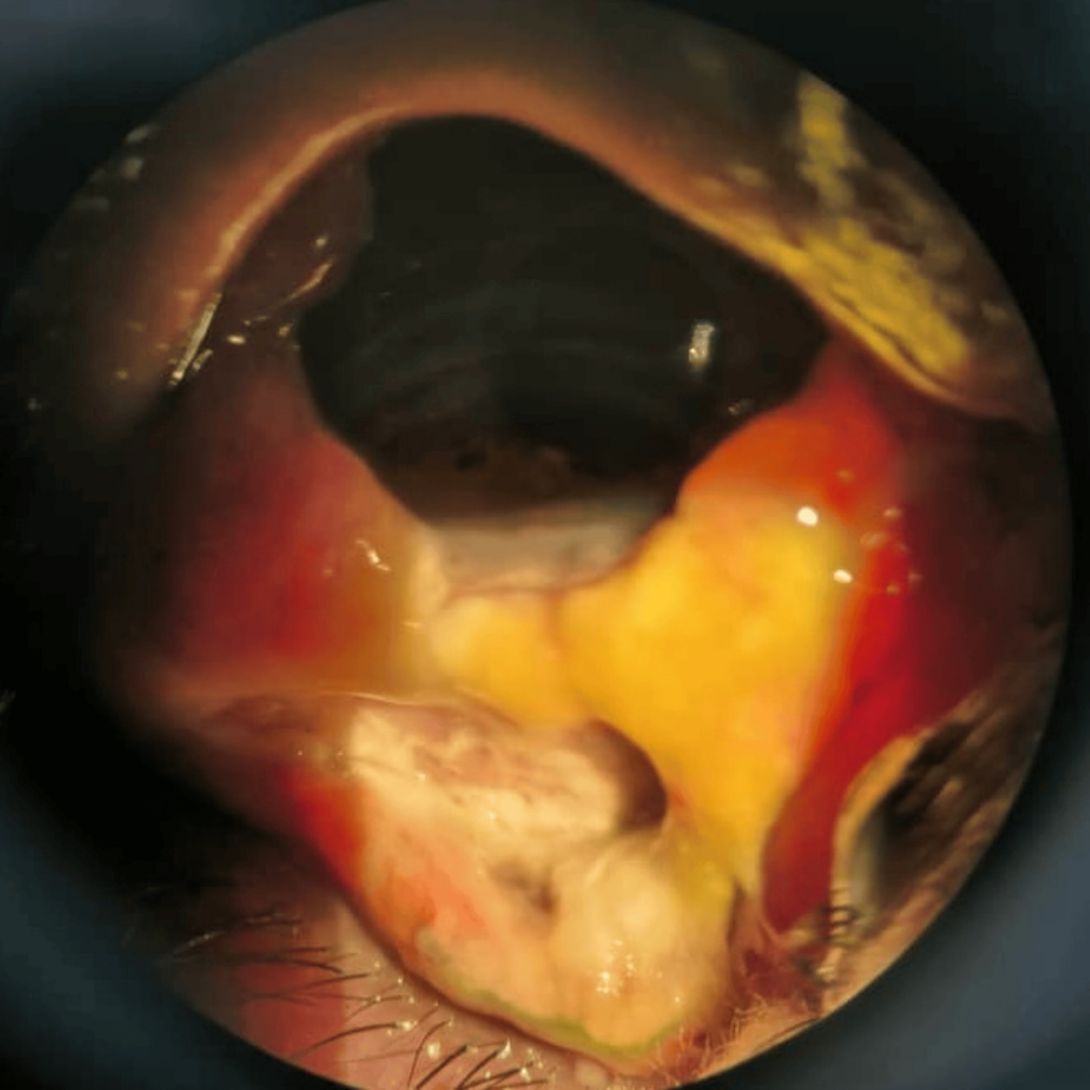
One week after percutaneous sclerotherapy for right orbitofacial venous malformation, showing extensive inferior scleral necrosis with subconjunctival abscess

**Figure 5:**
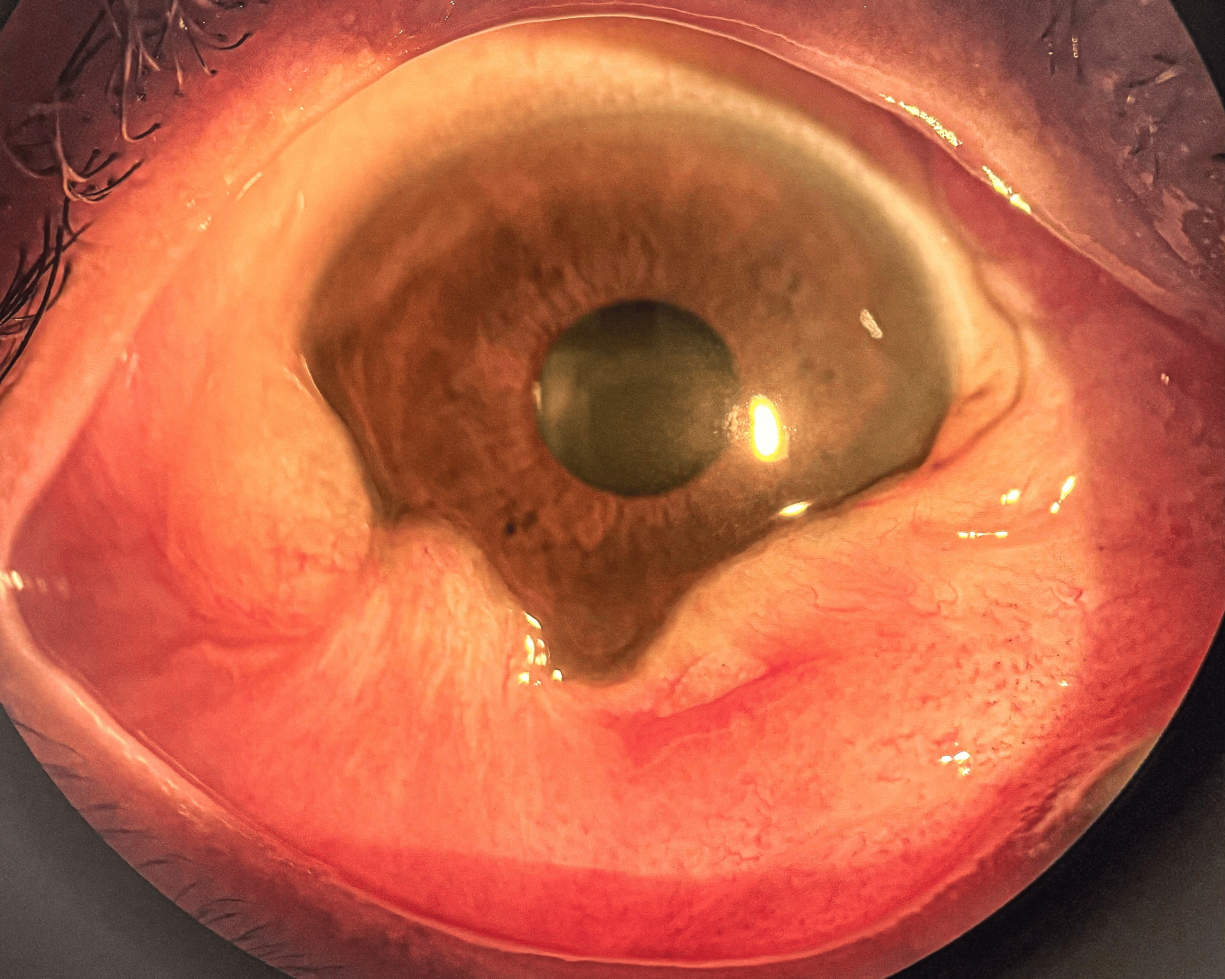
One month post-treatment for necrotizing scleritis, showing marked reduction in conjunctival swelling and redness, with scleritis under control

One week post-procedure, she reported a progressive increase in pain, swelling, and whitish ocular discharge in the RE, accompanied by a decline in visual acuity to 3/60. Slit-lamp examination demonstrated extensive scleral necrosis inferiorly, in conjunction with a subconjunctival abscess, confirming a diagnosis of necrotizing scleritis in the RE (Figure 4). A conjunctival swab was sent for microbiological culture, and she was treated empirically with intensive topical antimicrobial (moxifloxacin) to prevent secondary bacterial infection, along with oral nonsteroidal anti-inflammatory drugs (NSAIDs) - ibuprofen and doxycycline. The culture subsequently grew *Staphylococcus aureus*, which was sensitive to chloramphenicol. Topical chloramphenicol was added to her treatment regimen. Her condition gradually improved over the course of a month. RE chemosis reduced, and no sign of scleral thinning was seen (Figure 5). Her vision improved to 6/18, which was better than her baseline vision.

## Discussion

Orbitofacial VMs are rare, congenital vascular anomalies that arise from abnormal development of venous structures within the orbit and surrounding facial tissues. They typically occur in young to middle-aged individuals and show no gender predilection [2]. These lesions are generally slow-growing and low-flow. Their clinical presentation is variable, ranging from asymptomatic cosmetic disfigurement to significant functional impairment, including proptosis, diplopia, ocular motility restriction, or visual disturbances, particularly as they expand in response to changes in venous pressure. Orbital involvement is particularly challenging given the confined anatomical space and proximity to critical neurovascular structures. Imaging modalities, such as magnetic resonance imaging (MRI) and contrast-enhanced computed tomography (CT), play an essential role in delineating the extent of the lesion and guiding management.

Treatment strategies may involve observation, sclerotherapy, surgical excision, or a multimodal approach depending on lesion size, location, and functional impact. Percutaneous image-guided sclerotherapy is a well-established and safe treatment for orbitofacial VM. It works by occluding abnormal vessels and inducing vascular atrophy, especially in non-distensible, low-flow lesions, thereby allowing prolonged sclerosant activity [2]. Commonly used sclerosants include ethanol, pingyangmycin, sodium morrhuate, ethanolamine, STS, picibanil (OK-432), and polidocanol. STS is considered one of the milder sclerosants with a more favorable safety profile [3]. It is an anionic surfactant solution containing 2% benzyl alcohol, which induces endothelial cell necrosis and thrombus formation in abnormal vessels [4].

Despite having a favorable safety profile, several studies reported complications following percutaneous sclerotherapy for orbitofacial VM. The most common complications are mild and transient, such as local inflammation and swelling, which typically do not require special management. Major complications, though rare, are more frequently associated with potent sclerosants such as ethanol. These include vision loss, orbital compartment syndrome requiring emergent decompressive surgery, central retinal artery occlusion, nerve injury, and anaphylactic shock. However, there have been no previous reports of infective necrotizing scleritis following percutaneous sclerotherapy [5].

The development of necrotizing scleritis post-sclerotherapy is likely multifactorial, involving a combination of inflammation, vascular damage, and ischemia, which may compromise blood flow and ultimately lead to necrosis [6]. Additionally, the use of sclerosants may lead to tissue breakdown, potentially creating a portal of entry for pathogens. de los Mártireset al. [7] reported that various microorganisms can cause surgically induced infectious scleral necrosis, with bacterial infections being more common than fungal. Frequently isolated bacteria include *Pseudomonas aeruginosa, Staphylococcus aureus, Staphylococcus epidermidis, Streptococcus pneumoniae*, and *Mycobacterium *spp. Bacterial proteoglycan-degrading enzymes can break down collagen fibrils and the surrounding proteoglycan matrix [7]. 

Managing infectious scleritis is particularly challenging due to the sclera’s avascular nature, which limits drug penetration and contributes to delayed diagnosis. Prompt identification and targeted treatment are critical to prevent severe sight-threatening complications, such as scleral perforation, spread of infection, or the need for enucleation or evisceration. Management typically includes aggressive treatment with combination antimicrobial therapy and cautious immunosuppressive therapy after infection has been adequately controlled. While awaiting microbiological confirmation, empirical treatment with broad-spectrum fortified antibiotics targeting both Gram-positive and Gram-negative bacteria should be promptly initiated. A commonly used empirical regimen includes fortified cefazolin 5% along with ciprofloxacin 0.3% or tobramycin 0.3% eye drops [8]. The antibiotic regimen should then be adjusted based on preliminary smear findings and refined further according to culture and sensitivity results. Several empirical regimens have been described, with the most favorable outcomes typically achieved through a combination of topical and systemic antibiotics [8-10]. In severe cases, intravenous antibiotics may be necessary to ensure adequate scleral penetration. Clinical response is typically expected within 24-48 hours of starting treatment. Lack of improvement should prompt a reassessment and consideration of alternative therapies.

Corticosteroid therapy should be used with caution and close monitoring, as it may impair wound healing [11] and contribute to scleral thinning [9]. In our case, a corticosteroid was not prescribed due to the positive bacterial culture. Instead, an oral NSAID - ibuprofen - was administered, which effectively controlled the inflammation. Tappeiner et al. [12] and Foster et al. [13] proposed a stepwise management approach beginning with systemic NSAIDs, followed by systemic corticosteroids and then non-corticosteroid systemic immunomodulatory agents. As an adjunctive treatment, we also included oral doxycycline (100 mg/day) in the regimen for its anti-collagenolytic properties [14].

The patient’s infective necrotizing scleritis improved with treatment over the course of one month. This case underscores the critical importance of early recognition and prompt management of sclerotherapy-associated complications, which can significantly improve the prognosis.

## Conclusions

This report describes a rare case of infective necrotizing scleritis following percutaneous sclerotherapy, successfully managed with topical antibiotics and oral nonsteroidal anti-inflammatory therapy. The awareness of this vision-threatening complication is critical to facilitate early diagnosis and prompt intervention, thereby minimizing the risk of irreversible ocular damage.
